# Light extraction from fundamental modes in modulated waveguides for homogeneous side-emission

**DOI:** 10.1038/s41598-018-27916-x

**Published:** 2018-06-22

**Authors:** Zhiwen Pan, Lothar Wondraczek

**Affiliations:** Otto Schott Institute of Materials Research, University of Jena, Fraunhoferstrasse 6, 07743 Jena, Germany

## Abstract

Dedicated control of axial light emission from light-guides enables a new generation of functional light sources for volumetric illumination. A primary challenge is to ensure homogeneous emission intensity across the full length of the device. Here, we introduce an approach towards homogeneously side-emitting waveguides which do not rely on imposing local scattering centers such as bubbles, micro-/nanoparticles, and rough or abrupt interfaces, but on modulated core radius. Previous quantitative studies of the relationship between structural parameters and radiation losses provide initial conditions for tailoring side-emission through core-diameter modulations, however, with strongly limited amplitude of modulation. We now employ and verify numerical simulation to overcome this limitation towards meter-long homogeneously side-emitting waveguides in which the amplitude of core-diameter modulation is of the same order of magnitude as the core diameter itself. Similar emission properties can be obtained through modulation of the core refractive index instead of the core diameter, or through a combination of both approaches. Using the present model, we deduce exemplary conditions for homogeneous side-emission in which the power flow within the waveguides decays linearly, what may present another interesting feature for applications beyond illumination.

## Introduction

In optical waveguides, radiation loss through side-emission is normally undesired. On the other hand, besides active side-emission^1^, also passively side-emitting waveguides^2^ present an interesting approach for volumetric and functional lighting, for example, in biomedical treatment^3,4^ or to illuminate photochemical reactors^5^, provided that homogeneous lateral emission can be achieved.

Currently, one-dimensional (fiber) or two-dimensional (plate) illumination sources are applied in, *e.g*., indoor decoration^6^, agriculture^5,7^, water disinfection^8^ and photodynamic therapy^3,4,9^. Most of these applications, however, do not rely on homogeneous side emission alongside the full length of the waveguide, but on emission at the end or at individual spots across the waveguide. To achieve a homogeneous intensity distribution alongside a waveguide device of one or more meters in length, an axially invariant surface scattering function is required intuitively. But this produces an exponentially decaying power flow inside the waveguide, thus, exponentially decaying side-emission. Homogeneous relative radiation loss alongside the waveguide would be accompanied by linearly decaying power flow inside of the device. Therefore, either the properties of the waveguide material or the scattering function must be varied, leading to transversally non-uniform optical fibers.

Dielectric waveguides with slight imperfections are used to induce scattering of light out of the guidance regime. These imperfections include nanoparticulate precipitates, rough or abrupt surfaces or local refractive index variations. Dedicated tailoring of such scattering centers is possible only within strict technological limitations. Furthermore, problematic to real-world application, micro-bubbles, scratches or inclusions may significantly reduce the mechanical performance of the final device^10^, what prevents many of the potential applications.

**Figure 1:**
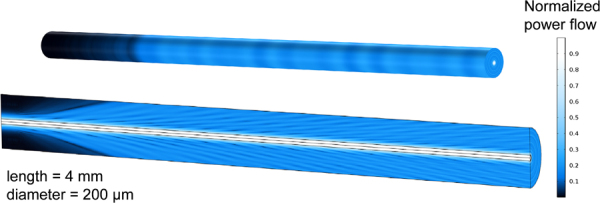
Schematic of a side emitting fiber with sinusoidal modulation of the core diameter, obtained from a separate finite element simulation on the starting section with Gaussian incident beam profile (approximate LP_01_ mode). The radiation loss from the core is illustrated through the normalized axial power flow in the fiber. The rings surrounding the cladding layer show the magnitude of axial Poynting vector arriving on the coating-cladding interface.

Another approach to introduce radiation losses from a waveguide is, therefore, axial modulation of the core radius, for example, in sinusoidal form (shown schematically in Fig. 1). The quantitative relationship between the modulation parameters (amplitude and period) and radiation loss has been studied by Marcuse^11^ and Snyder^12^, using the coupled-mode theory (CMT). The equations derived from CMT can only be solved for small modulations by employing the perturbation method, for example, for roughness-induced radiation losses from the fundamental mode in slab-waveguides and step-index fibers (it is noteworthy that any finite modulation function can be expanded into sinusoidal form by finite Fourier sine transformation. Thus, studying the case of sinusoidal modulation does not compromise on generality). However, for the perturbation solution to be valid, the amplitude of the modulations is required to be much smaller than the core radius, *e.g*., 50 nm for a core-radius of 5 µm. At such magnitude, modulations fall within the scale of surface roughness, and are difficult to tailor with high precision.

We now present a numerical study of larger sinusoidal modulations in core radius, with the modulation amplitude exceeding 5% of the average core radius. We employ the finite difference beam propagation method^13^ (FDBPM) to simulate beam propagation along symmetric slab-waveguides with sinusoidal core diameter modulations, and subsequently evaluate the radiation loss indirectly. The Hankel transform beam propagation method^14^ (HTBPM) is used to simplify the simulation of the propagation of a beam along a step-index fiber from 3D to 2D. We then take Marcuse’s perturbation solution (Eq. (79) in ref^15^. for slab waveguide and Eq. (4.4–13) in ref.^11^ for step index fiber) as analytical reference to validate our models. Using the validated models, we evaluate the radiation losses from fundamental modes due to large modulations on core radius of the waveguides. In addition, we evaluate also sinusoidal modulations on core refractive index. This provides an alternative parameter to tailor the radiation loss. Fabrication of the latter such modulations is possible up to 5 × 10^−3^ in magnitude^22^, in analogy to writing fiber Bragg gratings (FBG) using beam interference with a mask^16^ or point-by-point writing^17^.

## Model Construction and Assumptions

In our approach, we initially limit our discussion to weakly-guiding single mode fiber, similar to Marcuse’s perturbation solutions^11,15^. This enables to neglect the effects of polarization, and also to employ scalar homogeneous Helmholtz equations for the description of beam propagation. Then, the Helmholtz equations lead to paraxial wave equations by introducing the slowly varying envelope approximation in FDBPM. However, such treatment is not necessary for the HTBPM, in which an average reference refractive index is used instead of the effective refractive index of the propagation mode. Both numerical models and the analytical reference are restricted to real propagation constants, meaning that evanescence or leaky modes are not included in our discussion. Furthermore, the core medium has no absorption and both core and cladding medium are free from Rayleigh scattering, therefore, our result underestimates experimental expectations. As assumed in Marcuse’s reference solutions, both the forward and backward radiation losses^11^ from a forward-propagating mode are considered, while the radiation losses from a backward-propagating mode are weak and therefore neglected. The present numerical reference is consequently built on a one-way beam propagation algorithm. Both models evaluate waveguides supporting only fundamental modes (TE_1_ even mode for a 2D slab waveguide and LP_01_ mode for step index fiber). Besides the simplicity in calculation, the absence of other higher order guiding modes prevents complicated energy exchange. It therefore becomes easier to tailor the radiation losses. Furthermore, it seems safe to assume in this case that no energy transfer occurs from a radiation mode back into the fundamental mode (line 11-12 in page 114 in ref.^11^). Table 1 summarizes all assumptions which were used in the following.

**Table 1:** Assumptions of numerical models and the perturbation solution of CMT.

Assumptions	Marcuse’ perturbation solution of CMT	FDBPM	HTBPM
Slowly varying envelope	used	used	not used
Weak coupling	used	not used	not used
Weakly guiding	used	used	used
Real propagation constants	used	used	used
Only forward propagation	used	used	used
Only fundamental mode	used	used	used
No abruption discontinuity in refractive index in z direction: ∂*n*^2^/∂*z* ≈ 0	not used	used	not used

**Figure 2:**
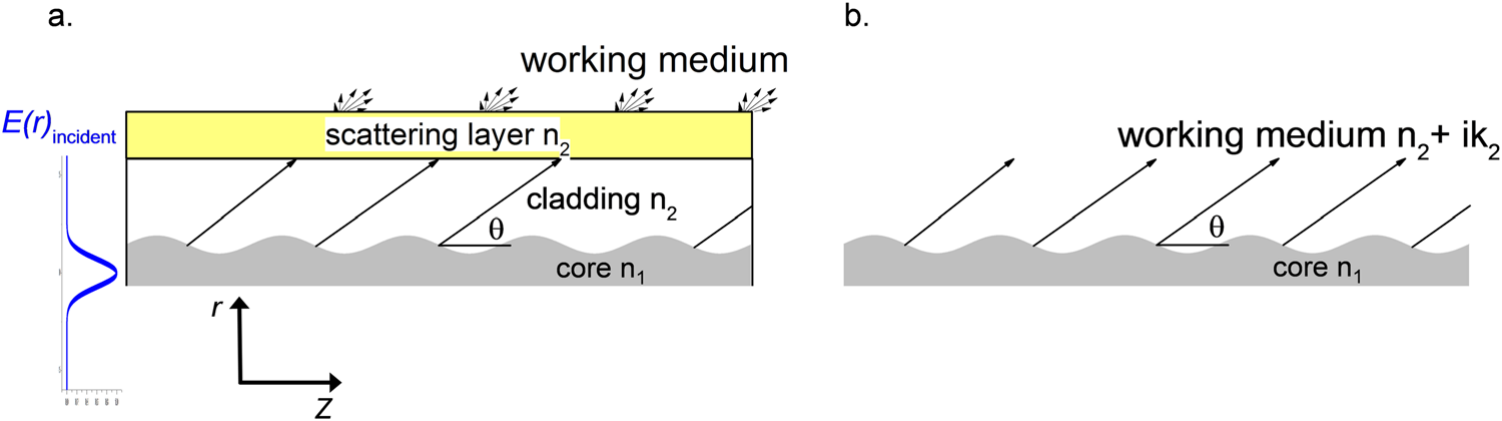
Schematic of possible working situations, corresponding to waveguides with infinitely large cladding as illumination sources. Environmental illumination is achieved via radiation losses due to sinusoidal modulation of the core radius. (**a**) Application in air or a medium with significantly lower refractive index than the core. (**b**) Application in a working medium whose refractive index is a bit lower than that of the core. In the latter case, the medium itself acts as an effective cladding, forming a step-index waveguide.

It should be noted that the radiation loss discussed in this paper accounts only for the light escaping from the core, which is equivalent to the presumption in the reference solutions that the cladding is infinitely large. Such an assumption is still valid when we consider the following practical situations shown in Fig. 2. If the waveguide is working in a medium with distinct refractive index (*e.g*., in air or water), a scattering layer (e.g., polymer coating, slightly phase separated/crystallized glass) with a somewhat higher refractive index can be attached to the exterior of the cladding (Fig. 2(a)). The radius of the cladding is chosen so that it is big enough for the fundamental mode to vanish on the boundary. The radiation arriving in the interior boundary of the coating scatters forward to the outside. The backscattered radiation can be safely ignored if we keep the mechanism of scattering within the Mie regime; *i.e*., the dimension in effective radius of the scattering centers is between half to about 300 times of the incident wavelengths. The scattering layer is only required to provide a constant scattering ability. Figure 2(b) demonstrates another working situation in which the refractive index of the working medium, *n*_2_, is just slightly lower than the core refractive index *n*_1_. In this case, neither a cladding nor an auxiliary scattering layer would be necessary in theory. Even though the radiation fields coming out of the periodic core interfere in the far field region, there is no obvious interference (fringes) in the near field, that is, in the vicinity of the scattering layer, to be confirmed in the following sections. The set-up shown in Fig. 2(b) corresponds to applications such as light sources for aquaponics and agriculture or water disinfection, in which the surrounding lossy medium absorbs the radiation in the near field so that no far field interference occurs. Under these considerations, the interference effect can be ignored in our discussion.

### Modelling homogeneous side emission

Since the power flow in waveguides consists of guiding and radiating contributions, constant transversal radiation loss (homogeneous side-emission) translates into a linear decay of the guided power *P*_*0*_ over the length *L*. Then, the propagating power *P(z*) along position *z* has the form

1 $$P(z)={P}_{0}\frac{L-z}{L}$$

**Figure 3:**
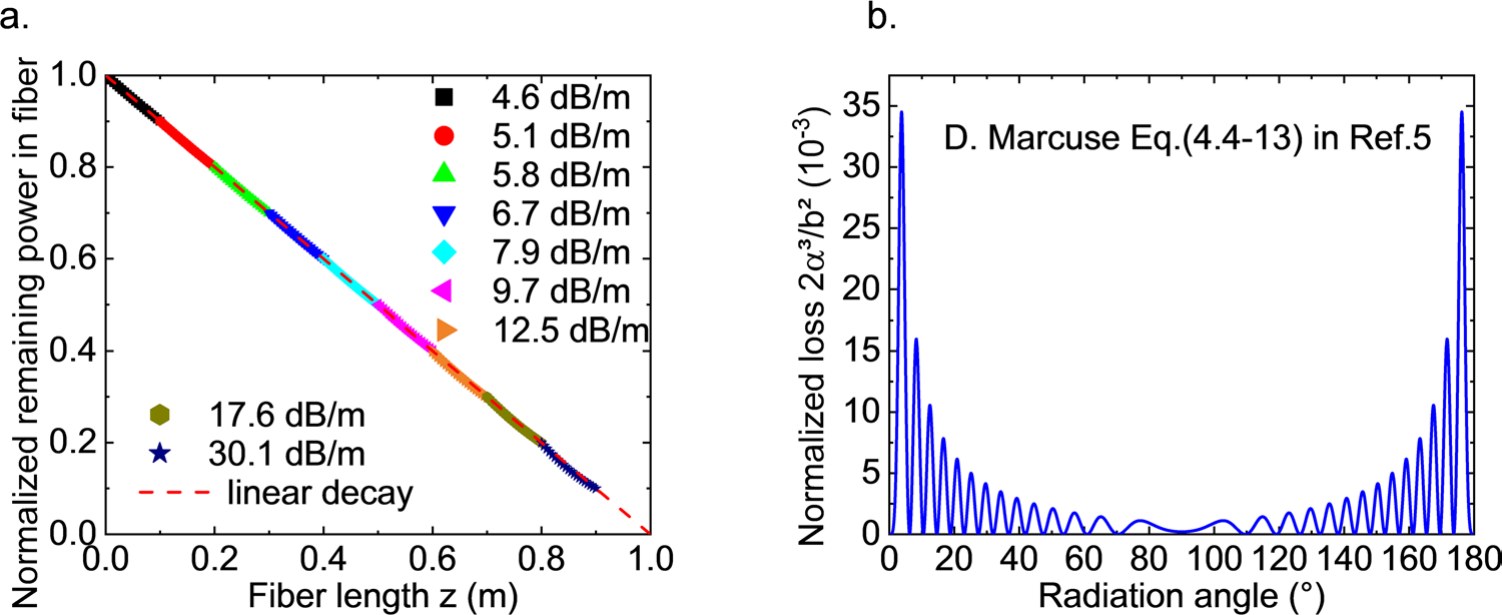
(**a**) Exemplar sequence of attenuation coefficients for a one-meter-long side-emitting fiber with transversally homogeneous emission intensity. The sequence comprises of 10 sections with varying periods of sinusoidal modulations. (**b**) Normalized loss coefficient as evaluated using the perturbation solution from CMT, Eq. (4), with core refractive index n_1_ = 1.460, cladding refractive index n_2_ = 1.458, incident wavelength λ = 1 µm, core amplitude b = 0.0474 µm and core radius a = 5 µm.

Constructing a waveguide consisting of *M* groups of periodic structures at intervals [*z*_*m*_, *z*_*m+1*_], *m* = 0, 1, 2… *M*, the power propagating within each group decays exponentially,

2 $$P({z}_{m+1})=P({z}_{m})\,\exp (\,\,-\,{\alpha }_{m}(z-{z}_{m}))$$

where *α*_*m*_ is the attenuation constant of the group *m*. Combining Eqs (1) and (2), the attenuation constant *α*_*m*_ for each group to approximate linear decay is

3 $${\alpha }_{m}=\,\mathrm{ln}(\frac{L-{z}_{m}}{L-{z}_{m+1}})/({z}_{m+1}-{z}_{m})$$

where *z*_*m*_, *z*_*m*+1_*, P*(*z*_*m*_) and *P*(*z*_*m*+1_) are known parameters. Figure 3(a) shows an example of a set of scattering losses to approximate a linear decay function using 10 sections of exponential decay by varying from 4.6 dB/m to about 30.1 dB/m according to Eq. (3). It is shown that using ten sections would be sufficient (with *R*^2^ = 0.999971 for the linear fit over position 0 to 0.9 m in Fig. 3(a)) to approximate linear decay (whereby the last section is always ignored because the exponential decay never reaches zero, while the linear decay does). The result can be easily extended to any other length *L* if the same amount of sections (e.g. 10 sections) are desired, although Eq. (3) seems to depend on *L*. If we set *z*_*m*_ to *cL*, where *c* is the fraction of length (0 < *c* < 1), the *L* in Eq. (3) cancels each other and the variation of *α*_m_ depends only on *M* (the number of sections).

As pointed out by Marcuse^5^ and Snyder^6^, any imperfection in core radius or refractive indices initiates energy exchange between any of the guided and radiation modes. If the relationship between attenuation constant *α* and modulation parameters is quantitatively known, our main objective is reached. Here, we take step index fibers as example (for slab waveguides, one may start from Eq. (79) in ref.^15^). The radiation loss which occurs in a single mode fiber due to a sinusoidal modulation of the core radius has been studied by Marcuse. The perturbation solution of the attenuation coefficient is given as Eq. (4.4–13) in ref.^11^:

4 $$2\alpha =\frac{1}{2}\pi {b}^{2}\frac{|{\beta }_{\varphi }|}{{\rho }_{\varphi }}\sum _{\mu ,p}{|{K}_{\mu 0}^{(p,+)}|}^{2}=\frac{1}{2}\pi {b}^{2}\frac{|{\beta }_{\varphi }|}{{\rho }_{\varphi }}\,({|{K}_{00}^{(p,+)}|}_{x\to x}^{2}+{|{K}_{20}^{(p,+)}|}_{x\to x}^{2}+{|{K}_{00}^{(p,+)}|}_{x\to y}^{2}+{|{K}_{20}^{(p,+)}|}_{x\to y}^{2})$$

where *α* is the attenuation constant for the field amplitude (therefore, 2α for power attenuation), *b* is the amplitude of the sinusoidal modulation, and *β*_*ϕ*_ is the propagation constant of the radiation mode at angle *ϕ* to the fiber axis. The *ρ* parameter is defined in Eq. (2.4–14) in Ref.^11^. *K* denotes coupling coefficients from the LP_01_ mode to forward (*p* = 1) and backward (*p* = 0) radiation modes, and *μ* is the azimuthal symmetry of the radiation mode. The subscript *x* → *x* indicates coupling from *x* to *x* polarized radiation modes while *x* → *y* means coupling from *x* to *y* polarized radiation modes. It is found that only the first term in the sum of mode square of *K* (representing coupling between identical polarization without azimuthal variation) is significant. The other three terms are neglectable. Eq. (4) is a perturbation solution at the limit of small amplitude of modulations as compared to the average core radius. The attenuation constants are evaluated at axial position far from a single period of modulation. Figure 3(b) shows one exemplar relation between normalized attenuation constants (valid for small modulation limit) and radiation angles *Θ*, which are correlated to periods of modulation *Λ* via Eq. (4.4–11) in ref.^11^. The reproduced curve in Fig. 3(b) is oscillating and symmetric, which differs from practical observation of random perturbation in the core radius as shown in Fig. 2(a) of Mazumder *et al*.^18^. The reason for this is that in CMT, the fundamental mode couples only to one single radiation mode with specific radiation angle due to the fact that only one spatial frequency exists after the Fourier transformation of the profile with fixed *Λ* (Eq. (4.4–13) in ref.^11^.). In practice, for a core radius profile with random perturbation, the Fourier transform of the core radius profile contains a range of spatial frequencies. This results in coupling to a broad range of radiation angles. Notwithstanding Rayleigh scattering, Fig. 3(b) shows the scattering loss at different specific radiation angles due to different structures with single spatial frequency. Figure 2(a) in ref.^18^, on the other hand, shows angle-dependent scattering losses due to a specific core radius profile with broad spatial frequencies after Fourier transform and, therefore, exhibits a broadened peak for the scattering losses. In practice, the modulation function will result from superposition of the desired sine modulation and a random modulation due to surface roughness. Therefore, practical measurement may hardly observe the oscillating results as the broadening effect will smoothen the curve as in Mazumder’s work.

The radius modulation can be expressed as

5 $$r(z)=a+b\,\sin (2\pi z/{\rm{\Lambda }}),$$

where *r(z)* is the core radius at axial position *z*, *b* and *Λ* are the amplitude and period of the sinusoidal modulation, respectively. The thus reproduced curve in Fig. 3(b) is exactly corresponding to Fig. 4.4.2 on page 159 of ref.^11^, where the ratio of *b:a* is kept at ~1%. For more pronounced modulation amplitude, this means that the single-mode condition can hardly be maintained. For example, a relevant practical scenario for side-emission would be to have *a* and *b* in the micrometers range, *e.g*., *b* = 1 μm, *a* = 10 μm, resulting in *b:a* = 10%. In this case, Eq. (4) breaks down. To extend the approach to large modulation amplitude, we now utilize beam propagation methods to solve the problem numerically. It will be shown that Eq. (4) is acceptable for amplitudes of modulation which do not exceed 5% of the average core radius. Otherwise (for example, at 10%), the reference solutions overestimate radiation losses especially at periods of modulation where maximum losses are predicted.

**Figure 4:**
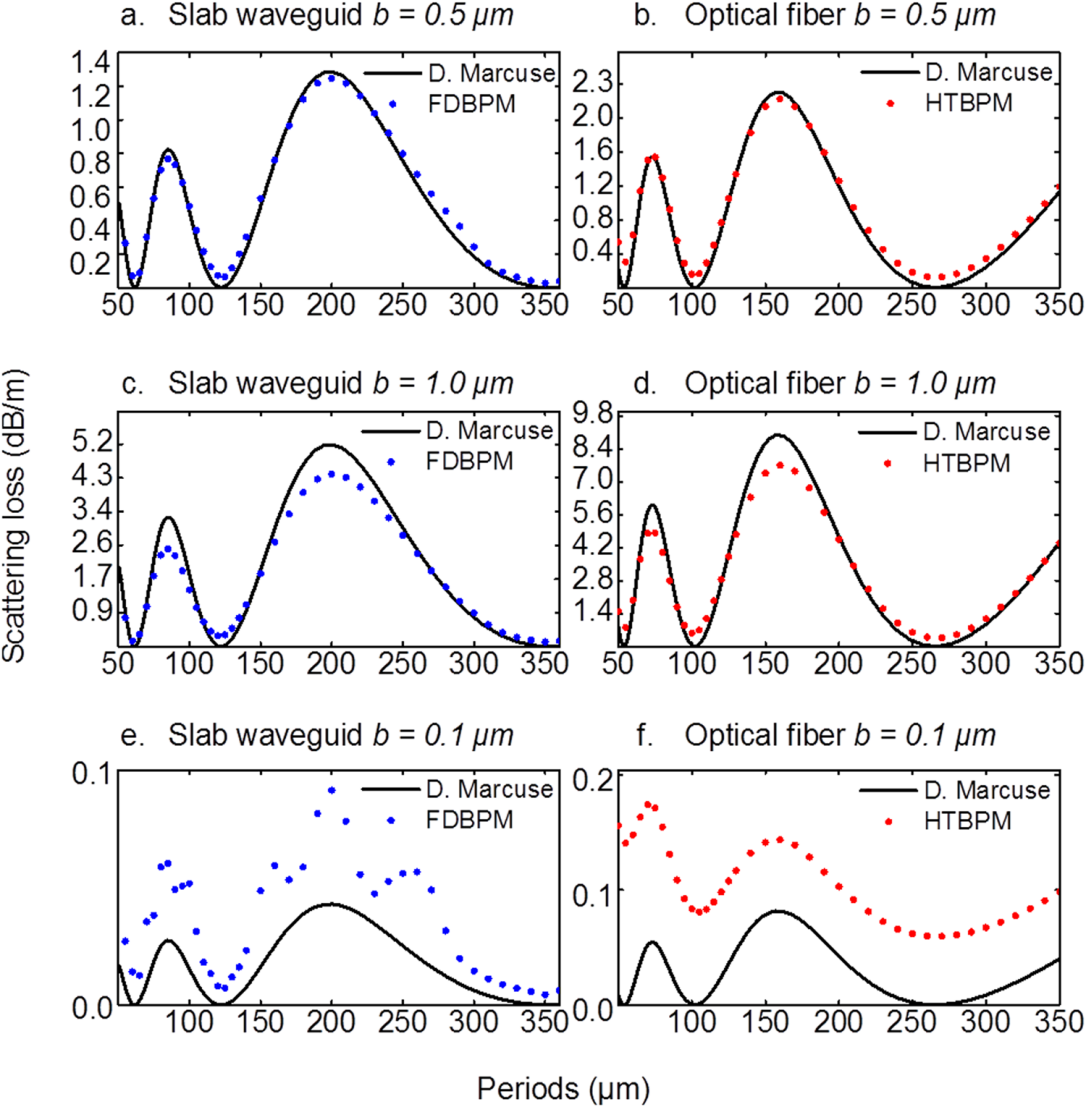
Evaluation of attenuation constants for radiation losses from the fundamental mode due to modulations on core radius with different modulation parameters, i.e., period Λ and amplitude *b*. The black solid line represents the analytical result using Eq. (4). Dotted lines in (**a**) (**c**) and (**e**) were obtained by FDBPM for symmetric slab waveguides with amplitude of modulation at 0.5 µm, 1.0 µm and 0.1 µm, respectively. Dotted lines in (**b**) (**d**) and (**f**) derive from HTBPM for step index fibers with amplitude of modulation at 0.5 µm, 1.0 µm and 0.1 µm, respectively. All calculations used refractive index *n*_1_ = 1.460, cladding refractive index *n*_2_ = 1.459, incident wavelength *λ* = 1.55 μm, core modulation amplitude *b* = 1 µm and core radius *a* = 10 µm. Further, *dr* = 0.001 µm, *dz* = 0.1 µm for FDBPM and *dz* = Λ/1000 for HTBPM.

### Verification of the BPM models

Figure 4 displays scattering losses evaluated by BPM models and by Marcuse’ perturbation solution. All methods evaluate radiation losses from the fundamental mode due to sinusoidal modulation of the core radius in slab waveguides (TE_1_ incident mode) and optical fibers (LP_01_ incident mode). The amplitudes of modulations are 1.0 µm, 0.5 µm and 0.1 µm over an average core radius of 10 µm. This corresponds to 10%, 5% and 1% modulations, respectively. Figure 4(a,b) show the best agreement between BPM simulations and the analytical results, with only minor deviations at the positions local maxima and minima. The deviation at maximum positions arises from the weak coupling assumption in the derivation of Marcuse’s perturbation method (Eq. 3.4–1 in ref.^11^), which assumes that the energy coupled to the radiation mode is so weak that the incident field does not change its shape and amplitude. This assumption is not valid when the coupling becomes stronger, resulting in an overestimation of radiation losses (the energy of the incident beam is still considered constant even though it decreases significantly). A similar deviation of local maxima is also seen in Fig. 4(c,d) where the amplitude *b* is twice in magnitude as compared to Fig. 4(a,b). According to Eq. (4), the radiation loss is proportional to the square of *b*, therefore, stronger overestimations arise in the perturbation solution shown in Fig. 4(c,d).

The deviations at minimum positions are similar in magnitude. They occur from numerical errors. Figure 4(e,f) show the radiation losses at 1% modulation of the core diameter. In this case, taking the analytical result as reference, the two BPM models exhibit different behavior in their numerical errors: random fluctuations and a bias for FDBPM and HTBPM, respectively. The source of errors lies in discretization, the paraxial approximation, and the reflection from the boundaries even though transparent boundary condition and absorption layers have been employed. Further tackling these problems seems unnecessary because the analytical perturbation solution holds at the limit of small modulations.

Practically, FDBPM differs significantly from HTBPM (2D FDBPM and 3D HTBPM). However, both methods show similar trends in Fig. 4(a–d), confirming that the numerical results are reliable and the overestimation from the analytical perturbation solution of the mode-coupling theory is not occasional.

## Results and Discussion

**Figure 5:**
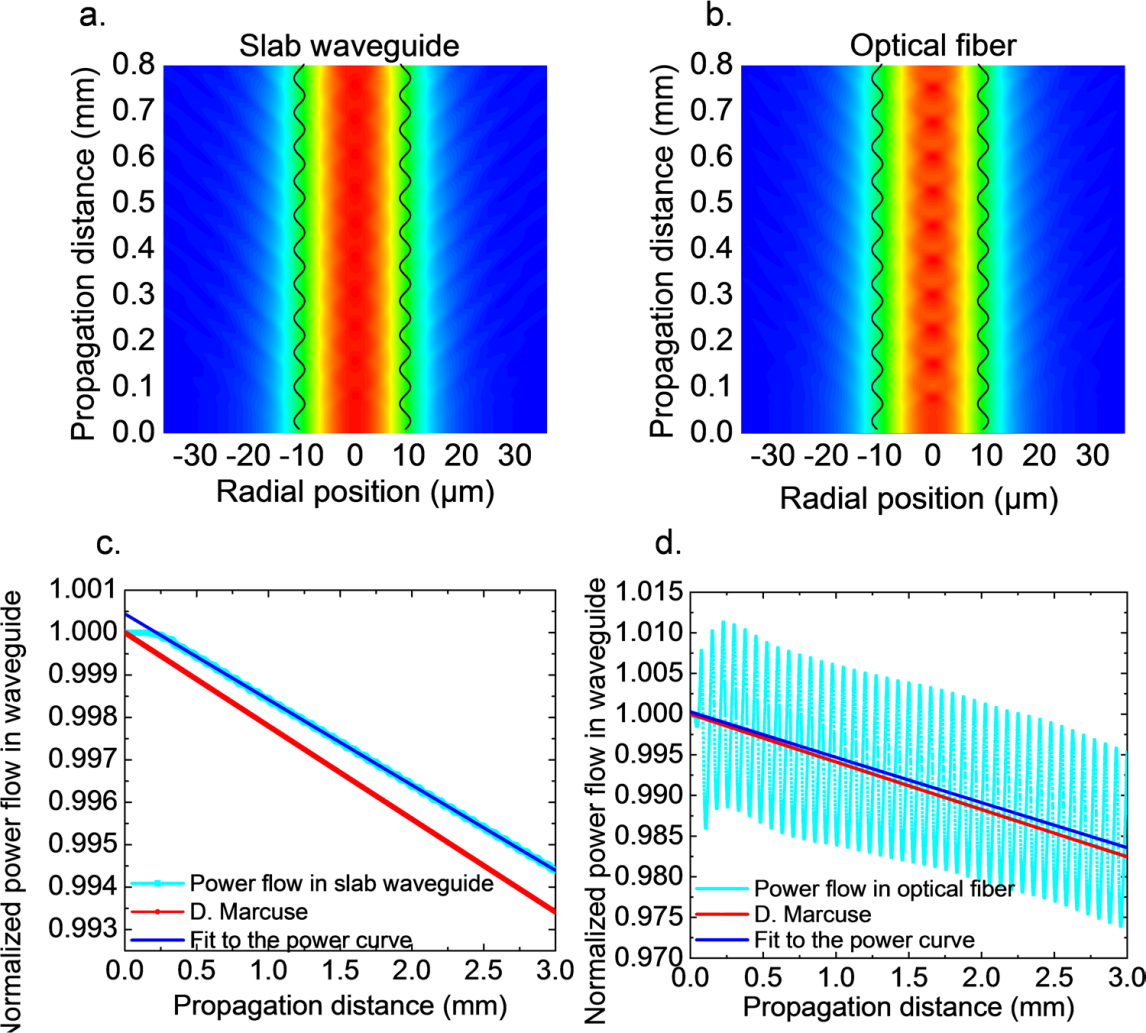
Normalized field distribution of a beam propagating in modulated core radius waveguides. (**a**) Symmetric slab waveguide with TE_1_ even incident mode from the bottom. (**b**) Step index fiber with LP_01_ incident mode from the bottom. (**c**) Corresponding axial power flow within the calculation window in (**a**) for a slab waveguide, simulated using slowly varying envelope approximation. (**d**) Corresponding axial power flow within the calculation window in (**b**) for a step index fiber simulated without using slowly varying envelope approximation. Calculations were done for core refractive index *n*_1_ = 1.460, cladding refractive index *n*_2_ = 1.459, incident wavelength *λ* = 1.55 µm, core modulation amplitude *b* = 1 µm and core radius *a* = 10 µm, *dr* = 0.01 µm, *dz* = 0.1 µm, Λ = 75 µm, *L* = 3 mm.

Figure 5 shows the normalized field distribution and normalized power propagation of a beam with the fundamental mode incident in a slab waveguide and a step index fiber, respectively. According to Fig. 5(a,b), the radiation loss occurs mainly at positions where the radius is narrowing in both slab waveguide and optical fiber. We chose a study window from −35 µm to 35 µm in radius for the integration of power flow to account for the confined power flow in the waveguide. Any fields outside the left and right boundaries in Fig. 5(a,b) are considered as radiation losses. By fitting the power flow within the study window, we estimate the attenuation constants using an exponential decay function with a single attenuation constant as shown in Fig. 5(c,d). The exponential decay function with attenuation constant predicted by the perturbation solution is plotted for comparison. For a period of *Λ* = 75 µm and a modulation amplitude of 1 µm, the perturbation solution overestimates the analytical result (Fig. 4(d)). The difference in power flow between Fig. 5(c,d) arises from the slowly varying envelope assumption in FDBPM, which is not employed in HTBPM for optical fiber, therefore, fast oscillations appear in Fig. 5(d).

### Exemplar conditions for homogeneously side-emitting fiber

In the design shown in Fig. 3(a), ten discrete values of attenuation constants ranging from 1 to 7 m^−1^ (4.6 to 30.1 dB/m) are required to generate approximately linear decay in guided power and, correspondingly, laterally homogeneous side-emission. Since one attenuation constant may correspond to several possible spatial periods of modulations, it is, in principal, possible to choose any period together with a certain amplitude of modulation. However, notwithstanding technical issues in the manufacture of waveguides, if periods of modulation are chosen over a wide range, also the radiation angles deviate over a wider range, what compromises emission homogeneity. We therefore prefer to select periods of modulation in an as-narrow-as-possible range. As shown in Fig. 3(b), when it is far from 90°, there are eight peaks occurring between 0° to 40°, averaging in a spacing of 2.5° among each half peaks. This indicates that if we choose different periods (in Fig. 4) of modulation distributed within half a peak, the biggest deviation in radiation angle (radiation angle in Fig. 3(b) is equivalent to period in Fig. 4) is just 2.5°. Here, we take a step index fiber with an amplitude of modulation of *b* = 1 µm as an example. It provides a scattering loss of up to 39.1 dB/m, covering the required range for a one-meter-long homogeneously side-emitting fiber. As shown in Fig. 4(d), there are two possible half peaks within which the modulation period can be chosen: [110 µm, 150 µm] and [170 µm, 240 µm]. The former one covers a range of 40 µm while the latter one covers a range of 70 µm. From the viewpoint of sensitivity, the former one produces a higher spatial resolution (due to smaller dimension), but would also require higher precision (due to smaller range) in the fabrication of periodic structures. In the example shown in Table 2, we therefore use the latter window of modulations.

**Table 2:** Exemplar design parameters of a one-meter-long homogeneously side-emitting waveguide.

Fiber sections (cm)	Period of sinusoidal modulation in core radius (µm)		Period of sinusoidal modulation in core refractive index (µm)		Attenuation constant (dB/m)
	Slab waveguide	Step index fiber	Slab waveguide	Step index fiber	
0–10	115.3	239.0	207.1	167.2	4.6
10–20	133.4	236.7	209.2	168.3	5.1
20–30	121.5	234.0	211.7	169.6	5.8
30–40	102.9	231.0	214.7	171.1	6.7
40–50	143.2	227.2	218.8	173.5	7.9
50–60	175.5	222.2	224.5	176.9	9.7
60–70	156.7	215.3	233.2	182.3	12.5
70–80	155.0	204.3	249.1	191.0	17.6
80–90	198.2	177.6	309.4	215.2	30.1
90–100	—	—		—	—

Average core radius: 10 µm.

Slab core height: 20 µm.

Core refractive index: 1.4600.

Cladding refractive index: 1.4590.

Amplitude of modulation in fiber core radius: 1 µm.

Amplitude of modulation in slab core thickness: 2.50 µm.

Amplitude of modulation in core refractive index: 5 × 10^−4^.

### Index versus radius modulation

In the previous sections, we demonstrated the ability of tailoring radiation loss by introducing core radius modulations at constant core refractive index. In an analogous approach, similar emission behavior can be generated by sinusoidal modulation of the core refractive index at constant core radius. This is of particular relevance when the modulations are not created through modifications of the fiber drawing process, but, *e.g*., through laser-assisted writing.

**Figure 6:**
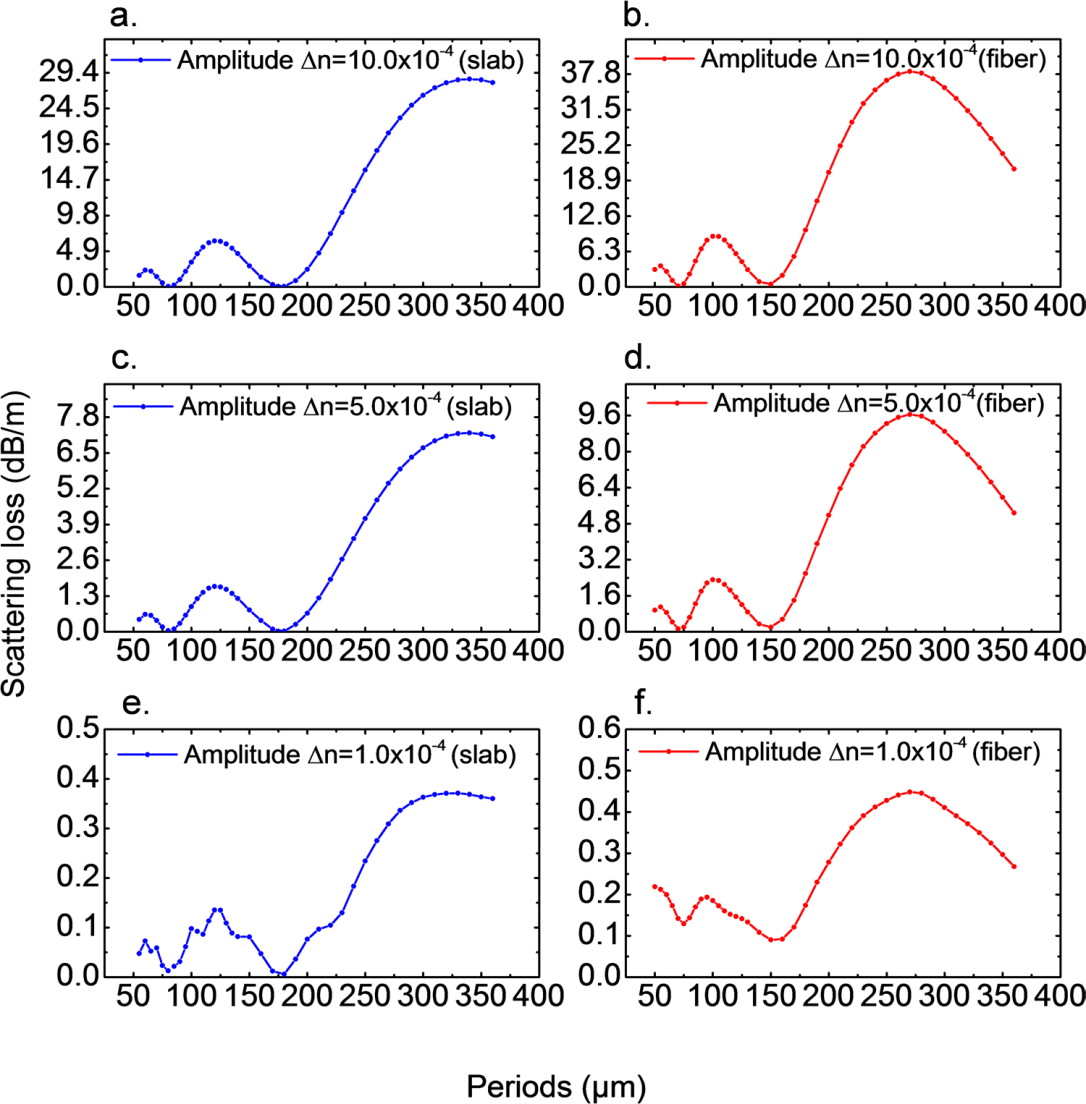
Simulated attenuation constants for radiation losses from fundamental modes due to axial modulation of the core refractive index. (**a**) (**c**) and (**e**) are simulated by FDBPM for symmetric slab waveguides with amplitude of modulation at Δ*n* = 10 × 10^−4^, Δ*n* = 5 × 10^−4^ and Δ*n* = 1 × 10^−4^, respectively. (**b**) (**d**) and (**f**) are simulated by HTBPM for step index fibers with amplitude of modulation at Δ*n* = 10 × 10^−4^, Δ*n* = 5 × 10^−4^ and Δ*n* = 1 × 10^−4^, respectively. All calculations are done for refractive index *n*_1_ = 1.460, cladding refractive index *n*_2_ = 1.459, incident wavelength *λ* = 1.55 μm, core modulation amplitude *b* = 1 µm and core radius *a* = 10 µm. *dr* = 0.001 µm, *dz* = 0.1 µm for FDBPM and *dz* = Λ/1000 for HTBPM.

Using the above-introduced BPM models, the radiation losses from fundamental modes which arise from such modulations of the core refractive index in slab waveguides and step index fibers are shown in Fig. 6. The requirement for a one-meter-long homogeneously side-emitting waveguide can be met by introducing an amplitude of modulation in refractive index of Δ*n* = 5 × 10^−4^ (Fig. 6(c,d)). Detailed design parameters for such a fiber are provided in Table 2. The radiation losses due to a modulation amplitude of Δ*n* = 1 × 10^−4^ are too low for a one-meter-long side-emitting waveguide. The curve shown in Fig. 6(a) is similar to 6(c) while the Fig. 6(b) is similar to (d). This similarity implies that a master curve as in Fig. 3(b) exists by dividing a same function of *b*, e.g. *b*^2^. When the radiation losses are extremely low (Fig. 6(e,f)), numerical errors occur which prevent the further applicability of the model.

**Figure 7:**
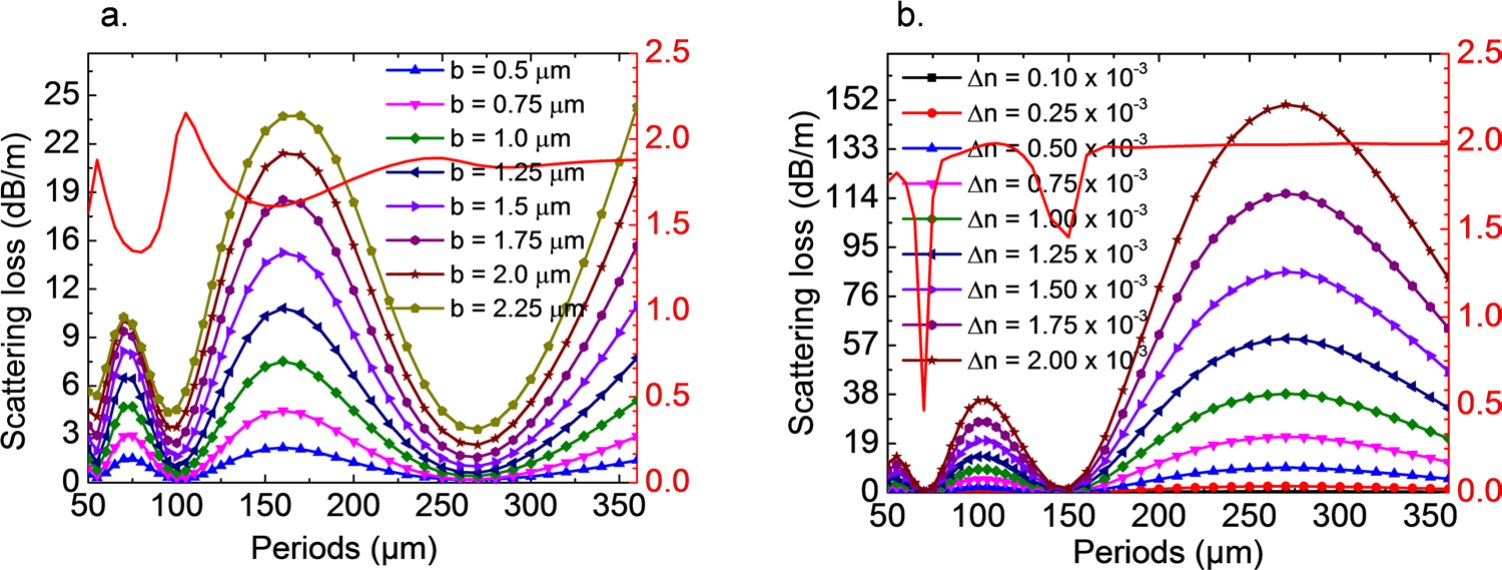
Simulated attenuation constants for radiation losses from fundamental mode due to different modulations on core radius (**a**) and on refractive index axially (**b**). The red curves in both cases represent simulated γ values, which are 2 in Marcuse’s perturbation solution for different periods of modulations. If it is smaller than 2 the attenuation constants develop due to increasing amplitude of modulation slower than the perturbation solution predicts and vice versa. The parameters used in the simulations are core refractive index *n*_1_ = 1.460, cladding refractive index *n*_2_ = 1.459, incident wavelength *λ* = 1.55 µm, core modulation amplitude *b* = 1 µm and core radius *a* = 10 µm. *dr* = 0.001 µm, *dz* = 0.1 µm for FDBPM and *dz* = Λ/1000 for HTBPM.

For the modulation of core radius, the radiation loss is proportional to the square of *b* (Eq. (4)) in the limit of validity of the perturbation solution. Figure 7(a) shows the radiation losses using the present numerical models with large modulation amplitudes in core radius, *i.e*., from 0.5 µm to 2.25 µm over an average radius of 10 µm. If we try to fit the radiation losses for different *b* in a power function, *b*^*γ*^, we find that γ is not a constant over varying periods, and that it deviates from 2, as shown in the red curve in Fig. 7(a). If *γ* < 2, there is a lower growth rate with increasing amplitude of modulation then predicted by the perturbation solutions. The lowest positions are corresponding to the maximum radiation losses, indicating the highest total overestimation. This was already discussed in the previous sections. The main reason for the deviation from 2 arises from the fact that the modulation amplitudes are large. If we follow the same process for the modulation in core refractive index, we get the results as shown in Fig. 7(b). The *γ* in this case is approximately 2 over most of the considered range of periods, except for the minimum positions, where numerical errors occur. This result implies that the amplitude of modulation in core refractive index is still within the limit of validity of analytical solutions. For the slab waveguides, similar observations were made (not shown).

As seen from Fig. 7, the modulation in core refractive index is more sensitive than the modulation of the core radius. The maximum radiation loss reaches  166 dB/m with an amplitude of modulation in refractive index of Δn = 20 × 10^−4^. Even when using small periods at about 50 µm, homogeneous side-emission can readily be achieved with an amplitude of Δn = 20 × 10^−4^. The advantage is that tailoring the core refractive index in a sinusoidal fashion is already well developed in the fabrication of fiber Bragg gratings using interferometric laser writing^16^. The period of the modulation in refractive index can be varied through the phase mask or the distance between fiber and mask. Then, the remaining technical requirement is to provide a low loss photosensitive material that is able to generate refractive index changes in the magnitude of 5 × 10^−4^, and its combination with a non- photosensitive cladding^19–22^.

## Conclusions

We presented the design of homogeneous side-emitting waveguides by introducing axial modulations in core radius or core refractive index. According to simulation data, these waveguides provide up to 33 dB/m for radius modulation and 166 dB/m for refractive index modulation in our calculation, compared to up to 10 dB/m in commercially available devices^2,6^. Available algorithms for calculating the quantitative relationship between radiation losses and modulation parameters were extended to large amplitudes of modulation via verified FDBPM for symmetric slab waveguides and HTBPM for step index fibers. We find good agreement between numerical models and the analytical perturbation solution from CMT when the amplitude of modulation in core radius is about 5% (relative to the core radius). Above 5% the radiation losses grow with increasing amplitude of modulation at different rates (predicted by the analytical perturbation solution), depending on the period of modulation. For modulation in core refractive index, we find that it is sufficient to use 0.03% modulation (relative to the core refractive index) to achieve a one-meter-long homogeneous side-emitting waveguide. Finally, we deduce exemplar conditions for homogeneously side-emitting one-meter-long slab waveguides and step index fibers using modulation in core radius as well as modulation in core refractive index. The presented model also applies without the assumptions of single-mode guidance, zero absorption, zero Rayleigh scattering and no backward propagation. Initial use of such assumptions only simplifies the case result validation.

## Appendix

### Finite Difference Beam Propagation Method

Beams propagation in the present case occurs mainly in axial direction. Such directional and not fast-spreading beams allow for a plane wave (paraxial wave) approximation and therefore can be modelled with an envelope function multiplied by a plane wave function:

6 $${{\bf{E}}}_{{\bf{t}}}={{\boldsymbol{\Psi }}}_{{\bf{t}}}\exp (\,-\,i{n}_{0}{k}_{0}z),$$

where ***E***_***t***_ is the electrical transversal field vector, ***Ψ***_***t***_ is the transversal envelope vector, *k*_*0*_ = *2π/λ* is the wavenumber (*λ* is wavelength of the beam), *n*_0_ is the reference refractive index and *z* is the axial position. With the weakly guiding approximation, substituting Eq. (6) into a homogeneous scalar Helmholtz wave equation leads to the paraxial wave equation

7 $${\rm{\Delta }}\phi -2i{n}_{0}{k}_{0}\frac{\partial \phi }{\partial z}+{k}_{0}^{2}({n}^{2}-{n}_{0}^{2})\phi =0,$$

where *φ* is a scalar envelope function representing either *Ψ*_*x*_ or *Ψ*_*y*_ in ***Ψ***_*t*_ and *n* is the local refractive index. Eq. (7) is the starting equation for both FDBPM and HTBPM. If ∆*φ* is expanded in cartesian coordinates for two-dimensional waveguides ($\partial /\partial y=0$) as assumed in FDBPM, Eq. (7) becomes

8 $$\frac{{\partial }^{2}\phi }{\partial {x}^{2}}+\frac{{\partial }^{2}\phi }{\partial {z}^{2}}-2i{n}_{0}{k}_{0}\frac{\partial \phi }{\partial z}+{k}_{0}^{2}({n}^{2}-{n}_{0}^{2})\phi =0$$

If *n*_*0*_ is suitably chosen, e.g., the effective refractive index of the propagating mode, the envelope *φ* can be considered as the slowly varying amplitude function of the propagating mode while the fast oscillations are described by the *exp*(*−in*_*0*_*k*_*0*_*z*) term. We now express the slowly varying envelope approximation in the second derivative,

9 $$\frac{{\partial }^{2}\phi }{\partial {z}^{2}}\ll 2{n}_{0}{k}_{0}\frac{\partial \phi }{\partial z}$$

Eq. (9) signifies that the second derivative of the envelope is neglectable with respect to *z* and, therefore, Eq. (8) can be further simplified to

10 $$\frac{{\partial }^{2}\phi }{\partial {x}^{2}}-2i{n}_{0}{k}_{0}\frac{\partial \phi }{\partial z}+{{k}_{0}}^{2}({n}^{2}-{{n}_{0}}^{2})\phi =0$$

The FDBPM discretizes the envelope function ${\phi }_{n}^{m}$ at positions (*n∆x, m∆z*) with *n* = 1, 2, …, *N, m* = 1, 2, …, *M*. Following the Crank-Nicolson scheme^13^, Eq. (10) can be discretized into

11 $$\begin{array}{c}(1-\alpha )\frac{{\phi }_{n-1}^{m}-2{\phi }_{n}^{m}+{\phi }_{n+1}^{m}}{{({\rm{\Delta }}x)}^{2}}+\alpha \frac{{\phi }_{n-1}^{m+1}-2{\phi }_{n}^{m+1}+{\phi }_{n+1}^{m+1}}{{({\rm{\Delta }}x)}^{2}}-\frac{2i{n}_{0}{k}_{0}}{{\rm{\Delta }}z}({\phi }_{n}^{m+1}-{\phi }_{n}^{m})\\ +(1-\alpha ){{k}_{0}}^{2}[{({n}_{n}^{m})}^{2}-{{n}_{0}}^{2}]{\phi }_{n}^{m}+\alpha {{k}_{0}}^{2}[{({n}_{n}^{m+1})}^{2}-{{n}_{0}}^{2}]{\phi }_{n}^{m+1}=0\end{array}$$

Eq. (11) consists of *N* linear equations and *N* + *2* unknown variables, i.e., *N* ${\phi }_{n}^{m}$ and the boundaries ${\phi }_{0}^{m}$, ${\phi }_{n+1}^{m}$. To solve Eq. (11), two more boundary conditions are therefore required. For these, we take the transparent boundary condition^13^ in the 2D FDBPM. *N* equations and two boundary conditions all together form a tridiagonal system which can be solved iteratively using the Thomas method (Appendix B in ref.^13^). If the initial envelope function ${\phi }_{n}^{1}$ is known as incident, the envelope function ${\phi }_{n}^{m}$ at positions *m∆z* can be evaluated by solving Eq. (11) iteratively. The incident field is TE_1_ even mode using core thickness 2*r*_*0*_ = 20 µm, wavelength 1.55 µm, core refractive index 1.460 and cladding refractive index 1.459.

### Hankel Transform Beam Propagation Method

For step index fibers, it is suitable to expand Eq. (7) in cylindrical coordinates:

12 $$\frac{{\partial }^{2}\phi }{\partial {r}^{2}}+\frac{1}{r}\frac{\partial \phi }{\partial r}+\frac{1}{{r}^{2}}\frac{{\partial }^{2}\phi }{\partial {\varphi }^{2}}+\frac{{\partial }^{2}\phi }{\partial {z}^{2}}-2i{n}_{0}{k}_{0}\frac{\partial \phi }{\partial z}+{{k}_{0}}^{2}({n}^{2}-{{n}_{0}}^{2})\phi =0$$

where r is the radius, and *ϕ* is the azimuthal angel. Since we are interested only in the fundamental mode of step index fibers, the mode field is radially symmetric, $\partial /\partial \varphi =0$, meaning that any derivative with respect to the azimuthal angel vanishes. Eq. (12) reduces to

13 $$\frac{{\partial }^{2}\phi }{\partial {r}^{2}}+\frac{1}{r}\frac{\partial \phi }{\partial r}+\frac{{\partial }^{2}\phi }{\partial {z}^{2}}-2i{n}_{0}{k}_{0}\frac{\partial \phi }{\partial z}+{{k}_{0}}^{2}({n}^{2}-{{n}_{0}}^{2})\phi =0$$

As we know one of the solution of cylindrical Helmholtz equation is the Bessel function of the first kind (the coefficient of the Bessel function of the second kind must be zero in order to keep the field finite at zero position), the order of Bessel function is determined by the angle-dependent part of *φ* during separation of variables:

14 $$\frac{1}{{\rm{\Theta }}}\frac{{\partial }^{2}{\rm{\Theta }}}{\partial {\phi }^{2}}=-\,{\nu }^{2}$$

where ν is the order of the Bessel function of the first kind. Since we assumed that the propagation field is radially symmetric, ν = 0 and therefore the solution of Eq. (13) can be expanded into a zero-order Bessel function

15 $$\phi (r,z)=\sum _{n}{a}_{n}(z){J}_{0}({Z}_{n}\frac{r}{R}),$$

where *Z*_*n*_ is the minimum radial position at which *n* zeros of the Bessel function can be found, and *R* is size of the calculation window in *r* axis. The expansion coefficient *a*_*n*_ is given by

16 $${a}_{n}(z)=\frac{{\int }_{0}^{R}\phi (r,z){J}_{0}({Z}_{n}\frac{r}{R})rdr}{{\int }_{0}^{R}{J}_{0}^{2}({Z}_{n}\frac{r}{R})rdr}$$

To obtain Eq. (16), the orthogonal property of zero-order Bessel function is used

17 $${\int }_{0}^{R}{J}_{0}({Z}_{n}\frac{r}{R}){J}_{0}({Z}_{m}\frac{r}{R})rdr=\frac{{R}^{2}}{2}{J}_{1}^{2}({Z}_{n}){\delta }_{mn}$$

where *δ* is the Kronecker delta. According to the BPM technique, field propagation as described in Eq. (13) consists of two successive steps: first advances ∆*z* in a homogeneous medium with refractive index *n*_*0*_, followed by another step with a phase shift according to the refractive index variation. The first step can be realized in the expansion coefficient *a*_*n*_ by

18 $${a}_{n}(z+{\rm{\Delta }}z)={a}_{n}(z)\exp (\,-i{\rm{\Delta }}z\sqrt{{n}_{0}^{2}{k}_{0}^{2}-\frac{{Z}_{n}^{2}}{{R}^{2}}})$$

while the second step can be realized using

19 $$\phi (r,z+{\rm{\Delta }}z)=\phi (r,z)\exp (\,-\,i{\rm{\Delta }}z{k}_{0}n)$$

If the incident field $\phi (r,0)$ is known, it is first expanded according to Eq. (16) and then advances a step with Eq. (18) in the *a*_*n*_ domain. It is then possible to reconstruct the field using Eq. (15) with the coefficients obtained from Eq. (18), and finally the field is modulated by a phase term using Eq. (19). Repeating these steps, the fields at other positions can be obtained successively.

The transformation we used is mathematically the Hankel transformation, therefore this method is termed as HTBPM. The HTBPM has great advantage in computational expense, firstly, it reduces the 3D propagation in step index fiber to 2D propagation. If 3D FDBPM is used by introducing the ADI method^23^, the computational cost increases by several orders of magnitude. Secondly, only half of the radius domain is calculated. Besides, HTBPM does not use the slowly varying envelope assumption in its derivation as compared to FDBPM. For the boundary condition in HTBPM, we assume an absorbing boundary, in which a layer of 30 mm thickness contains a gradually increasing imaginary part of refractive index. The incident field is the LP_01_ mode of a structure with core radius of *r*_0_ = 10 µm, λ = 1.55 µm, core refractive index 1.460 and cladding refractive index 1.459.

### Data Availability Statement

The datasets generated during and/or analyzed during the current study are available from the corresponding author on reasonable request.
